# Predictive Analyses of Biological Effects of Natural Products: From Plant Extracts to Biomolecular Laboratory and Computer Modeling

**DOI:** 10.1093/ecam/nep096

**Published:** 2011-03-09

**Authors:** Roberto Gambari

**Affiliations:** Department of Biochemistry and Molecular Biology, Section of Molecular Biology, Via Fossato di Mortara, 74, 44100 Ferrara, Italy

## Abstract

Year by year, the characterization of the biological activity of natural products is becoming more competitive and complex, with the involvement in this research area of experts belonging to different scientific fields, including chemistry, biochemistry, molecular biology, immunology and bioinformatics. These fields are becoming of great interest for several high-impact scientific journals, including *eCAM*. The available literature in general, and a survey of reviews and original articles recently published, establishes that natural products, including extracts from medicinal plants and essential oils, retain interesting therapeutic activities, including antitumor, antiviral, anti-inflammatory, pro-apoptotic and differentiating properties. In this commentary, we focus attention on interest in networks based on complementary activation and comparative evaluation of different experimental strategies applied to the discovery and characterization of bioactive natural products. A representative flow chart is shown in the paper.

## 1. Plant Extracts Exhibit Biological Properties Relevant for Alternative Treatments of Human Diseases

**Figure 1:**
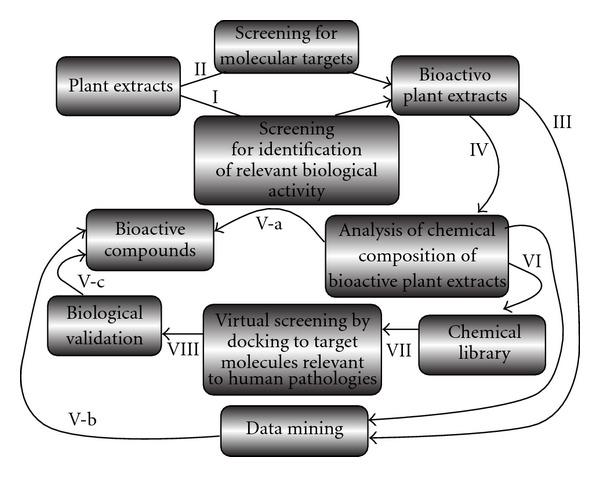
The interplay between several actions contributing to the characterization of biological activity of natural products from plant extracts. Numbering (I–VIII) facilitates the presentation of the different activities leading to the final identification of bioactive compounds starting from plant extracts, through intermediate products (databases from bioactive plant extracts, chemical libraries).

A first level of screening that is followed by many research groups allows the identification of plant extracts and essential oils exhibiting relevant biomedical effects (action I of the flow chart shown in Figure 1) [1–8]. A careful review of the conclusions in available literature reveals that several plant extracts exhibit activity against herpes simplex virus [4]. On the other hand, several plant extracts stimulate expression of differentiation-related functions of great value for developing drugs against important genetic diseases [9].

A complementary approach focusing on mechanism(s) of action instead of bio-medical effects identifies possible molecular targets of plant extracts (action II of Figure 1). Lampronti et al. [10] were able to demonstrate that extracts from medicinal plants differentially inhibit molecular interactions between nuclear factor *κ*B (NF-*κ*B) and target DNA. The finding of activity against a transcription factor (TF) stimulated experiments on effects of single plant extracts or essential oils on biological functions depending on this TF. NF-*κ*B is deeply involved in inflammatory processes, as well as antiapoptotic properties. Hence, plant extracts inhibiting NF-*κ*B (for instance, extracts from *Emblica officinalis*) deeply modify the production of inflammation-related proteins induced in bronchial cell lines by *Pseudomona aeruginosa* (PAO) infection [11]. Nicolis et al. [11] demonstrated that extracts from *E. officinalis* strongly inhibited the PAO-dependent expression of the neutrophil chemokines IL-8, GRO-*α* and GRO-*γ*, of the Inter-Cellular Adhesion Molecule 1 (ICAM-1) and of the pro-inflammatory cytokine IL-6, when tested in human IB3-1 bronchial epithelial cells exposed to the *P. aeruginosa* laboratory strain PAO1. This finding might be relevant for treatment of the inflammatory process of cystic fibrosis (CF) [12]. In fact, the most important cause of morbidity and mortality in CF patients is the lung pathology characterized by chronic infection and inflammation sustained mainly by *P. aeruginosa*.

Penolazzi et al. [13] demonstrated that *E. officinalis* extracts induce apoptosis of primary osteoclasts (OCs), as a possible treatment of osteoporosis and rheumatoid arthritis. In this study, the effects of extracts from *E. officinalis* on differentiation and survival of human primary OC cultures obtained from peripheral blood were determined by tartrate acid-resistant acid phosphatase positivity and colorimetric studies based on 3-(4,5-Dimethylthiazol-2-yl)-2,5-diphenyltetrazolium bromide (MTT) assay. The effects of *E. officinalis* extracts on induction of OC apoptosis were studied using the terminal dUTP nick-end labeling (TUNEL) assay and immunocytochemical analysis of Fas receptor expression. Extracts of *E. officinalis* were able to induce programmed cell death of mature OCs, without altering the process of osteoclastogenesis. *Emblica officinalis* increased the expression levels of Fas, a critical member of the apoptotic pathway. Interestingly, the extracts of *E. officinalis* inhibited in both the cellular systems described by Nicolis et al. [11] and by Penolazzi et al. [13] the expression of the pro-inflammatory IL-6 gene when analyzed by reverse transcriptase-polymerase chain reaction (RT–PCR) and immunocytochemistry.

Effects of plant extracts on cytokines have been recently published in *eCAM* by Zhao et al. [14], Saad et al. [15] and Park et al. [16]. The use of botanicals in osteoarthritis and rheumatoid arthritis has also been proposed by Ahmed et al. [17].

## 2. Looking for Lead Compounds within Plant Extracts

After the formal demonstration of a relevant biological activity, including possible effects on specific molecular targets, two complementary approaches can be followed for identification of putative lead compounds. The first (action IV of Figure 1) is a direct chemical analysis of plant extracts, based on several methods such as gas chromatography/mass spectrometry (GC-MS) and high-performance liquid chromatography/MS (HPLC-MS). The second (action III of Figure 1) is an activity of data mining focusing on what is available in the literature concerning bioactive plant extracts. Both these approaches can generate sets of molecules that are possibly responsible for biological activity found in analyzed extracts (actions V-a and V-b of Figure 1). This is an important process that will ultimately help in identifying lead compounds to be employed in preclinical studies. Examples of this process are those reported by Nicolis et al. [11] who identified pyrogallol as the bioactive molecule within extracts of *E. officinalis* that inhibit IL-8 gene expression. 

More recently, Guerrini et al. [18] have found that citropten and bergapten, detected in epicarps of *Citrus bergamia* fruits, are powerful inducers of differentiation and *γ*-globin gene expression in human erythroid cells. These data could have practical relevance, since pharmacologically mediated regulation of human *γ*-globin gene expression, with the consequent induction of fetal hemoglobin, is considered as a potential therapeutic approach in hematological disorders, including *β*-thalassemia and sickle cell anemia [9].

## 3. Identification of Molecular Targets and Studies of Structurally Related Compounds

Collaboration between chemists, molecular and cellular biologists is of great added value. The identification of putative molecular targets is important for at least two reasons: (i) development of advanced biological assays and (ii) screening of sets of structurally related compounds. Several research groups have hypothesized specific cellular targets for lead compounds identified in medicinal plant extracts. For instance, Dat et al. [19] identified the phenolic constituents of *Amorpha fruticosa* that inhibit NF-*κ*B activation and related gene expression. When the identification of a molecular target is available, then novel approaches might be undertaken to characterize both mechanisms of action, binding modes and novel bioactive molecules (actions VI–VIII of Figure 1). Piccagli et al. [20] recently reported a docking study to NF-*κ*B-p50 on a data set of 27 molecules from extracts of two different medicinal plants. The purpose of the study was to develop a docking protocol fit for the target under study [20]. They enhanced the simple docking procedure by means of a sort of combined target- and ligand-based drug design approach. The results sustain the concept that docking performance is predictive of a biochemical activity. Through molecular docking simulations, this article represents an example of successful indentification, starting from a set of molecules found in plant extracts, of a lead compound promising for inhibition of NF-*κ*B-p50 biological activity and modulation of expression of NF-*κ*B-regulated genes (in this case the IL-8 gene) [20]. Similar computer-based strategies have been recently applied to other natural products, looking for effects on different biological functions by Rollinger et al. [21] on *Ruta graveolens* extracts, by Chen et al. [22] on suanzaroen decotion and by Paoletta et al. [23] using a data set from 240 herbs used in traditional Chinese medicine. These recent studies support the concept that computer-assisted approaches, such as pharmacophore modeling, virtual screening, docking and neural networking will help in identifying bioactive metabolites in extracts from medicinal plants [24]. Integrated computer-assisted strategies may help to process the huge amount of available structural and biological information in a reasonably short time for a straightforward search of bioactive natural products (action V-c of the flow chart shown in Figure 1) [24, 25].

## 4. Final Comments

Analyzing biological activity of plant extracts is rapidly moving from simple observations to integrated research approaches involving analytical chemistry, chemical synthesis, molecular and cellular biology, proteomics, transcriptomics and bioinformatics. The comparative analysis of complementary studies will bring novel information on bioactive compounds from natural products, including molecular targets and putative mechanisms of action. In particular, *in silico* tools, combined with classical research methods, are expected to be more frequently applied by natural product scientists in an effort to maximize efficacy in drug discovery. These multidisciplinary studies are expected to bring novel molecules of great biomedical relevance. The journal *eCAM* is deeply involved in bringing to the scientific community high-level relevant communications.
